# Mapping of Powdery Mildew Resistance Gene *pmCH89* in a Putative Wheat-*Thinopyrum intermedium* Introgression Line

**DOI:** 10.3390/ijms160817231

**Published:** 2015-07-28

**Authors:** Liyuan Hou, Xiaojun Zhang, Xin Li, Juqing Jia, Huizhen Yang, Haixian Zhan, Linyi Qiao, Huijuan Guo, Zhijian Chang

**Affiliations:** 1 College of Life Science, Shanxi University, Taiyuan 030006, Shanxi, China; E-Mail: houliyuan0131@163.com; 2 Institute of Crop Science, Shanxi Academy of Agricultural Sciences, Taiyuan 030031, Shanxi, China; E-Mails: zxjemail@163.com (X.Z.); leexinlee@aliyun.com (X.L.); 18636626922@163.com (H.Y.); zhan030006@126.com (H.Z.); qiaoly1988@126.com (L.Q.); ghj8067@126.com (H.G.); 3 Key Laboratory for Crop Gene Resources and Germplasm Enhancement on the Loess Plateau, Ministry of Agriculture, Taiyuan 030031, Shanxi, China; 4 College of Agronomy, Shanxi Agricultural University, Taigu 030801, Shanxi, China; E-Mail: jiajuqing@126.com

**Keywords:** wheat-*Thinopyrum intermedium* introgression line, *Th. intermedium*, powdery mildew, genetic analysis, molecular mapping

## Abstract

Powdery mildew, caused by *Blumeria graminis* f. sp. *tritici* (*Bgt*), is a globally serious disease adversely affecting wheat production. The *Bgt*-resistant wheat breeding line CH09W89 was derived after backcrossing a *Bgt* resistant wheat-*Thinopyrum intermedium* partial amphiploid TAI7045 with susceptible wheat cultivars. At the seedling stage, CH09W89 exhibited immunity or high resistance to *Bgt* pathotypes E09, E20, E21, E23, E26, Bg1, and Bg2, similar to its donor line TAI7045 and *Th. intermedium*. No *Th. intermedium* chromatin was detected based on genomic *in situ* hybridization of mitotic chromosomes. To determine the mode of inheritance of the *Bgt* resistance and the chromosomal location of the resistance gene, CH09W89 was crossed with two susceptible wheat cultivars. The results of the genetic analysis showed that the adult resistance to *Bgt* E09 in CH09W89 was controlled by a single recessive gene, which was tentatively designated as *pmCH89*. Two polymorphic SSR markers, *Xwmc310* and *Xwmc125*, were linked to the resistance gene with genetic distances 3.1 and 2.7 cM, respectively. Using the Chinese Spring aneuploid and deletion lines, the resistance gene and its linked markers were assigned to chromosome arm 4BL in the bin 0.68–0.78. Due to its unique position on chromosome 4BL, *pmCH89* appears to be a new locus for resistance to powdery mildew. These results will be of benefit for improving powdery mildew resistance in wheat breeding programs.

## 1. Introduction

Common wheat (*Triticum aestivum* L.) is the most widely cultivated and important staple food crop in the world, and is constantly challenged by many diseases such as powdery mildew and rusts. Powdery mildew, caused by *Blumeria graminis* f. sp. *tritici* (*Bgt*), is a globally serious disease of wheat. It often occurs in regions with cool and humid climates, resulting in severe yield reductions [1,2]. In China, especially in the southwestern region, powdery mildew is the most frequently occurring disease due to moderate temperatures and rainy conditions during the wheat growing season. Yield losses can surpass 100 million kg per year when powdery mildew epidemics occur [3]. However, powdery mildew pathogen populations are very dynamic due to the continuous appearance of new virulent pathotypes capable of overcoming widely used host resistance genes. As a result, many previously resistant wheat varieties become susceptible. Therefore, new sources of effective and durable resistance genes are required for breeding high-yielding cultivars. Selection of and field deployment of resistant varieties is the most economic, effective, and environmentally friendly approach to controlling the disease [4]. Thus, the discovery and utilization of new powdery mildew resistance genes have become common objectives for wheat geneticists and breeders worldwide.

Fifty-three formally designated major genes (*Pm1*-*Pm53*) [5,6,7] and over 100 quantitative trait loci (QTLs) [8,9] for resistance to powdery mildew have been identified on the 21 chromosomes in bread wheat. The majority (39) of them are derived from common wheat, and a minority (18) were transferred from wild relatives of wheat. *Thinopyrum intermedium* (Host) Barkworth and Dewey (2*n* = 6*x* = 42, JJ^s^S), an uncultivated relative of wheat, has many potentially useful agronomic characteristics that could be used in wheat breeding programs, such as wide adaptability and tolerance to cold, drought, and salinity.

*Th. intermedium* has been widely studied in research directed toward wheat improvement due to its immunity or resistance to many serious wheat diseases. Hence, a series of addition lines, substitution lines, and translocation lines have been developed over the past years and are available for both agronomic improvement and resistance breeding. To date, many multi-resistant lines have been developed by crossing susceptible wheat cultivars with resistant partial amphiploids as donor parents. So far, two powdery mildew resistance genes (*Pm40* [10] and *Pm43* [11]), one stem rust resistance gene (*Sr44* [12]), and one stripe rust resistance gene (*Yr50* [13]) have been transferred from *Th. intermedium* into common wheat. *Pm40*, *Pm43*, *Sr44* and *Yr50* have been mapped to chromosomes 7BS, 2DL, 7Dand 4BL, respectively.

CH09W89, a *Th. intermedium*-derived wheat introgression line, is highly resistant to powdery mildew. It exhibits resistance to powdery mildew under greenhouse conditions in Taiyuan, Shanxi province. We identified a number of wheat genotypes with effective resistance against common Chinese races. The purposes of the present study were to determine the resistance inheritance of and locate the new gene in the *Th. intermedium*-derived line CH09W89.

## 2. Results

### 2.1. Powdery Mildew Responses

**Figure 1:**
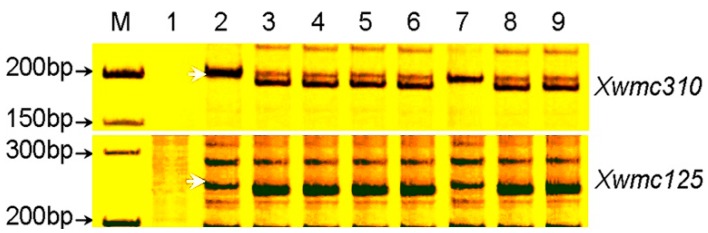
Silver-stained polyacrylamide gels showing simple sequence repeat (SSR) markers *Xwmc310* and *Xwmc125*. Lanes **1**: *Th*. *intermedium* Z1141; **2**: TAI7045; **3**: Jin T2250; **4**: Jinchun 5; **5**: Jinmai 33; **6**: Jintai 170; **7**: CH09W89; **8**: SY 95-71; **9**: Mianyang 11. Z1141, the accession of parent *Th. intermedium*; TAI7045, partial amphiploid and the resistant parent of CH09W89; Jin T2250 and Jinchun 5, the wheat parents of TAI7045; and Jinmai 33 and Jintai 170, the wheat parents of CH09W89. **M**: 100-bp DNA ladder. Arrows indicate polymorphic bands.

CH09W89, wheat-*Thinopyrum intermedium* partial amphiploid TAI7045, and the *Th. intermedium* parent Z1141 were resistant to all seven Chinese *Bgt* races tested at the seedling stage, whereas wheat parents Jinchun 5, Jin T2250, Jintai 170, and Jinmai 33 were susceptible (IT 3–4) (Table 1, Figure 1). The resistant ITs (infection types) were similar to the donor wheat-*Thinopyrum intermedium* partial amphiploid TAI7045 (IT 0–0;) as well as the donor *Th. intermedium* accession Z1141 (IT 0).

**Table 1:** Seedling infection types (IT) on selected donor lines, parents, and controls to seven *Bgt* pathotypes.

Line	*Bgt* Pathotype						
	E09	E20	E21	E23	E26	Bg1	Bg2
*Th. intermedium* Z1141	0	0	0	0	0	0	0
TAI7045	0	0	0;	0	0;	0	0
Jinchun 5 ^a^	4	4	4	4	3	4	4
Jin T2250 ^a^	4	4	3	4	4	4	4
CH09W89	0, 0	0	0	0	0	0	1
Jintai 170 ^b^	4	4	3	4	4	4	4
Jinmai 33 ^b^	4	4	4	4	3	4	4
SY95-71	4	4	4	4	4	4	4
Mianyang 11	4	4	4	4	4	3	4
Jingshuang 16	4	4	4	4	4	4	4

^a^ Wheat parent of TAI7045; and ^b^ wheat parent of CH09W89. Scores of 0–2 were classified as resistant and 3–4 as susceptible reactions.

### 2.2. GISH Identification of Alien Chromatin in CH09W89

**Figure 2:**
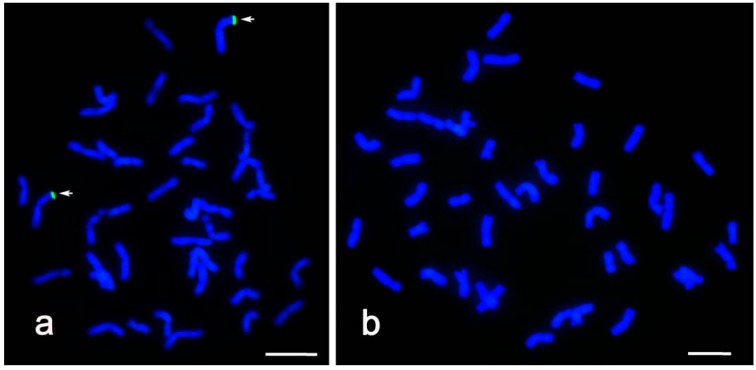
GISH pattern of lines 03W006, the wheat-*Thinopyrum intermedium* recombinant control (**a**) and CH09W89 (**b**) at mitosis using *Th. intermedium* genomic DNA as the probe. Arrows indicate alien chromatin. Scale bar = 10 μm.

GISH analysis was performed on somatic cells of both the resistant line CH09W89 and the recombinant control (line 03W006) using *Th. intermedium* genomic DNA as the labeled probe and Chinese Spring (CS) genomic DNA as blocker DNA. A pair of chromosomes revealed green fluorescence signals at the distal regions of their short arms in the positive control (Figure 2a). No visible hybridization signal could be found in CH09W89 (Figure 2b), indicating that no detectable wheat-*Th. intermedium* translocation is present in CH09W89. However, the translocated chromosome segment (if present) might be too small to be detected by GISH.

### 2.3. Inheritance of the Resistance to Powdery Mildew in CH09W89

At the adult plant stage, when inoculated with race E09, F_1_ plants from both crosses (SY95-71/CH09W89 and CH09W89/Mianyang) showed infection types (IT 3–4) similar to the susceptible parent, showing that the resistance gene was recessive (Table 2). Segregation in the F_2_ and BC_1_ populations (Table 2) included many plants with intermediate responses. When the numbers of F_2_ plants with IT 0–2 and those with IT 3–4 were pooled as separate resistant and susceptible groups, the ratios were consistent with those expected for segregation at a single locus. When tested with the same race, the F_2:3_ lines from SY95-71/CH09W89 and the BC_1_ plants from CH09W89/MY11//CH09W89 segregated one homozygous resistant (*HR*): two segregating (*Seg*): one homozygous susceptible (*HS*), and one resistant: one susceptible, respectively, confirming segregation ratios for a single gene (Table 2). In addition, in segregating F_3_ lines from SY95-71/CH09W89, the pooled numbers of resistant and susceptible plants were 1130 susceptible: 373 resistant (χ^2^_(3:1)_ = 0.03, *P*_1df_ = 0.87). These results suggested that a single recessive gene for resistance to powdery mildew was present in CH09W89, and was provisionally designated *pmCH89*.

**Table 2:** Adult plant segregation ratios of powdery mildew response in F_1_, F_2,_ and BC_1_ plants, and F_3_ lines when inoculated with *Bgt* isolate E09.

IT	Parent			P_2_/P_1_^a^					P_1_/P_3_	P_1_/P_3_//P_1_
				No. of Plants		No. of Lines			No. of Plants	No. of Plants
	P_1_	P_2_	P_3_	F_1_	F_2_	F_2:3_^b^			F_1_	BC_1_
						*HR*	*Seg*	*HS*		
0	15				11	9 ^c^	0	0		7
0	3				23	23	0	0		25
1					9	8 ^c^	0	0		4
2					3	3	0	0		1
3				3	78	0	62	16	5	24
4		16	17	15	51	0	14	31 ^d^	12	17
**Total**	**18**	**16**	**17**	**18**	**175**	**43**	**76**	**47**	**17**	**78**
				χ^2^ ^e^_(3:1)_ = 0.15		χ^2^_(1:2:1)_ = 1.37				χ^2^_(1:1)_ = 0.21
				*p* = 0.69		*p* = 0.50				*p* = 0.65

^a^ P_1_ = CH09W89, P_2_ = SY95-71, P_3_ = MY (Mianyang) 11; ^b^ *HR*, *Seg*, and *HS*: homozygous resistant, segregating, and homozygous susceptible; ^c^ Insufficient seeds were gained from these F_2_ plants due to very late heading. They were assumed to be *HR*; ^d^ Six F_2_ plants died in the greenhouse due to serious infection. They were assumed to be *HS* or *Seg*; ^e^ Values for significance at *p* = 0.05 are 3.83 for 1 *df* and 5.99 for 2 *df*.

**Figure 3:**
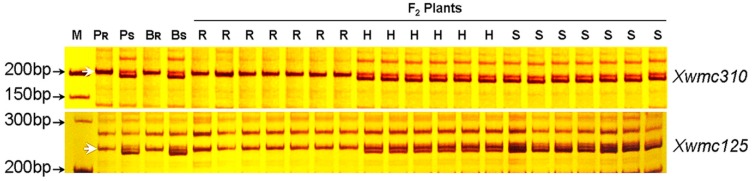
Silver-stained polyacrylamide gels showing simple sequence repeat (SSR) markers *Xwmc310* and *Xwmc125* flanking the *pmCH89* locus. *P_R_* and *P_S_* denote CH09W89 and SY95-71, respectively; *B_R_* and *B_S_* denote the resistant and susceptible bulks, respectively. Selected homozygous resistant (*R*), homozygous susceptible (*S*), and heterozygous (*H*) F_2_ plants from SY95-71/CH09W89 are included here. **M**, 100 bp DNA ladder; arrows on the left side indicate the fragment linked to the resistance gene. Arrows indicate the polymorphic bands.

### 2.4. Identification of Microsatellite Markers Linked to pmCH89

A total of 596 SSR markers, covering all 21 pairs of chromosomes, were used to screen polymorphisms between resistant (resistant parent and *Br* (bulked resistant)) and susceptible materials (susceptible parent and *Bs* (bulked susceptible)). About 156 (26.2%) of the microsatellite primers chosen for initial screening were polymorphic between resistant and susceptible materials. Four markers, *Xbarc193*, *Xbarc199*, *Xwmc125* (Figure 3), and *Xwmc310* (Figure 3), were associated with *pmCH89*. Linkage analysis using the four markers on F_2_ plants and the powdery mildew response genotypes inferred from reactions of the F_2:3_ families indicated that they were linked to the resistance gene. The three SSR markers, *Xwmc125*, *Xbarc199*, and *Xwmc310*, were located on the long arm of chromosome 4B, and *Xbarc193* on the short arm of chromosome 4B [14] (http://wheat.pw.usda.gov/cgi-bin/graingenes), indicating that *pmCH89* is located on 4B. The F_2_ population segregated 1:2:1 for all four markers.

**Figure 4:**
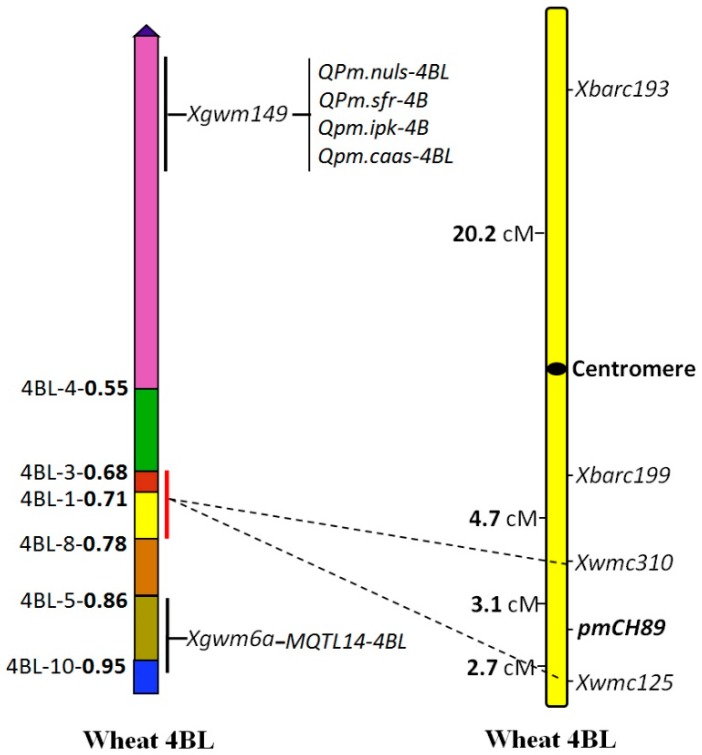
Genetic and deletion bin position of *pmCH89* on chromosome 4BL.

Analyses with Joinmap 4.0 also showed linkage between the markers and *pmCH89*; *Xwmc310* and *Xwm125* were close to the resistance gene with genetic distances of 3.1 and 2.7 cM, respectively, and *Xbarc199* and *Xbarc193* were more distant with respective genetic distances of 7.8 and 28.0 cM (Figure 4).

### 2.5. Chromosome Arm Assignment and Deletion Bin Mapping

**Figure 5:**
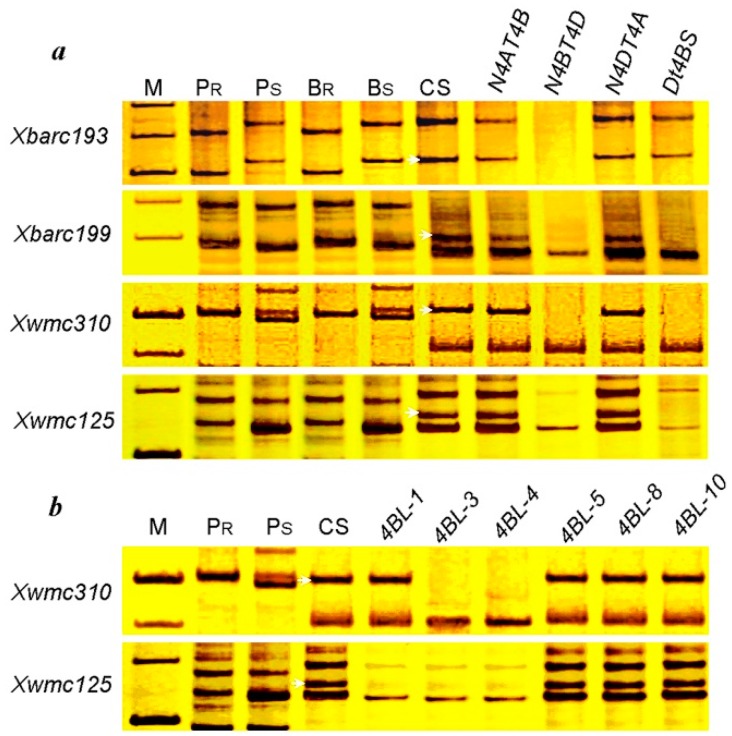
Amplification patterns of linked markers in CH09W89 (*P_R_*), SY95-71 (*P_S_*), resistant bulk (*B_R_*), susceptible bulk (*B_S_*), Chinese Spring (CS), nullisomic-tetrasomic (NT) 4B, ditelosomic (Dt) 4BS stocks (**a**); and 4BL deletion lines (**b**) of CS; Arrows indicate the critical bands.

To determine the location of *pmCH89* on chromosome 4B, the four SSR markers *Xwmc125*, *Xbarc193*, *Xbarc199*, and *Xwmc310* (http://wheat.pw.usda.gov/cgi-bin/graingenes) were used to test a set of Chinese Spring (CS) nullitetrasomic and ditelosomic lines. The four microsatellite primer pairs amplified products of the expected sizes in CS and the CS nulli-tetrasomic lines N4AT4B and N4DT4B, but no PCR product was observed for the nulli-tetrasomic N4BT4A and Dt4BS lines (Figure 5a), except *Xbarc193*. Only *Xbarc193* had PCR products in the ditelosomic line Dt4BS. Because amplified products of three markers were absent in the N4BT4A and Dt4BS lines, we confirmed the assignment of the linked microsatellite markers to the long arm of chromosome 4BL.

The deletion lines of CS chromosome 4BL were used to determine the physical bin locations of *pmCH89* and its flanking markers. *Xwmc310* and *Xwmc125* were not detected in 4BL-4 and 4BL-3, and *Xwmc125* was also absent in 4BL-1 (Figure 5b). The results indicated that *Xwmc310* is located at 4BL bin 0.68–0.71 and that *Xwmc125* is at 4BL bin 0.71–0.78. Therefore, the powdery mildew resistant gene *pmCH89* could be assigned to 4BL bin 0.68–0.78 (Figure 4). Based on its origin and map location, the recessive gene *pmCH89* is apparently new.

## 3. Discussion

Exploitation of genetic variability, especially for genes conferring disease resistance, is essential for the development of new, improved plant varieties. The uncultivated relatives of wheat have provided some of the most important and widely used resistance genes which have been deployed in commercial cultivars around the world. *Th. intermedium* is an important perennial Triticeae species for wheat improvement due to its resistance to viral and fungal diseases. Some wheat-*Th. intermedium* derivatives have been found to be highly resistant to the fungus causing powdery mildew. For example, a resistance gene was recently found in partial amphiploids [15] and a substitution line 2E (2D), in which an E-chromosome of *Th. intermedium* was substituted for chromosome 2D in wheat [16]. CH09W89, a putative derivative of *Th. intermedium* accession Z1141, was produced by crossing and backcrossing TAI7045, a partial wheat-*Th. intermedium* amphiploid, with susceptible wheat cultivars and selecting for powdery mildew resistance. Line CH09W89 displayed good agronomic characteristics and also carried resistance to powdery mildew. However, there was no documentation of the chromosome content of this line. As all of the wheat parents in the pedigree of CH09W89 are highly susceptible, the resistance in that line is assumed to derive from *Th. intermedium*. Genetic segregation data clearly indicate the presence of a single recessive resistance gene in CH09W89 (Table 2). Two other powdery mildew resistance genes, *Pm40* [10] and *Pm43* [11], previously introgressed into common wheat from this donor species, were dominant. Given its action and chromosomal location, the resistance gene in CH09W89 seems to involve a novel locus.

In general, because of the presence of the *Ph1* gene on wheat chromosome 5B, the recovery of a wheat-alien recombinant is difficult due to the lack of homoeologous pairing between the chromosomes of distant wild relatives and those of wheat. However, chromosomes of *Th. intermedium* and wheat can recombine [17,18], suggesting that the transfer of alien chromosome segments by spontaneous chromosome translocations from this species to wheat chromosomes is possible. A good example of this appears to be the current study in which a small *Th. intermedium*-derived segmental translocation carrying resistance to powdery mildew has apparently been produced by backcrossing the resistant partial amphiploid TAI7045, derived from *Th. intermedium*, with a susceptible wheat cultivar [19]. However, GISH analysis of CH09W89 produced no cytological evidence for an alien translocation in this study. No apparent linkage drag was observed in the introgression line CH09W89. The gene *pmCH89*, based on widespread effectiveness and a unique chromosome location, must be either present in an intercalary cryptic translocation from *Th. intermedium* or a wheat gene derived from an unknown source. Cryptic alien chromosome transfers have been reported in other studies [20,21,22]. Further studies are needed to confirm the source of *pmCH89*.

In the present study, *pmCH89* conferred full resistance at the seedling stage to seven different *Bgt* isolates (Table 1), and was mapped onto wheat chromosome arm 4BL with a different location from *Pm40* (7BS) [10] and *Pm43* (2DL) [11]. Among the currently designated and temporarily designated *Pm* genes, some of them confer recessive resistance. These include *Pm5* on chromosome 7BL, originally derived from *T. dicoccum* [7]; *Pm9* and *mlRD30* on chromosome 7AL [7], *Pm47* on chromosome 7BS, from *T. aestivum* [23]; *Pm26* [7] and *pm42* [24] derived from *T. dicoccoides* and mapped on chromosome 2BS; *pmY212* on chromosome 5DL, originating from *Ae. tauschii* [25]; *pm2026* on chromosome 5A^m^L, derived from *T. monococcum* [7]; *MlHubel* on chromosome 2DL, transferred from *T. spelta* [26]; and *PmLK906* and *pmX* [27], both located on chromosome 2AL, and derived from *T. aestivum* [7].

Several QTLs for adult plant resistance with major or minor effects on powdery mildew response were also mapped on chromosome 4BL in previous studies [8]. Among them, *QPm.nuls-4BL*, a major QTL with resistance contributed by Avocet, accounting for between 21.0% and 40.2% of the total phenotypic variation in a RIL population, was located on the region around *Xgwm251* and *Xgwm375* [28]. This QTL was at the same position as similar QTLs *QPm.sfr-4B*, *Qpm.ipk-4B*, and *Qpm.caas-4BL*, detected respectively in the Forno/Oberkulmer, Synthetic/Opata, and Fukuho-komugi/Oligoculm populations [8]. Their location is very close to the centromere of chromosome 4B due to the close linkage to *Xgwm149* [14], whereas in this study, *pmCH89* was physically mapped on the intercalary bin 0.68–0.78 of chromosome 4BL, about 3 cM proximal to *Xwmc125* (Figure 4 and Figure 5). This would seem to be a different locus. Additionally, Marone *et al.* [9] detected a major QLT, MQTL14-4BL, locus for powdery mildew resistance from *T. durum* on 4BL between the markers *Xbcd110* and *Xgwm6a*. This gene also has a different chromosomal location from *pmCH89* because the distal flanking marker *Xgwm6a* for MQTL14-4BL is proximal to *Xwmc125* with a genetic distance of 16 cM [14] and its physical location is in chromosome 4BL distal bin 0.86–1.00 [29].

In this current work, linkage analysis showed that a putative *Th. intermedium*-derived powdery mildew resistance gene *pmCH89* was flanked by *Xwmc310* and *Xwm125* (Figure 4). Between the flanking markers *Xwmc310* and *Xwmc125*, a genetic distance of 5.8 cM was found. This indicated that there was recombination between these loci as well as between them and the resistance gene. Interestingly, the markers, which span 5.8 cM in the SY95-71/CH09W89 cross, cover approximately 23 cM in the Synthetic/Opata cross [14]. This shows that the recombination rate in the present study is about three-fold lower than in a cross involving hexaploid wheat. These results suggest that pairing and crossovers between chromosome 4B and an unidentified fragment of *Th. intermedium* chromosome introgressed into CH09W89 seem to be possible but at a reduced rate. In fact, such suppressed recombination is common in populations segregating for alien introgressions in plants. For example, powdery mildew resistance genes *Pm12* and *Pm27*, introgressed into common wheat chromosome 6B from *Aegilops speltoides* and *Triticum timopheevii*, respectively, showed no recombination or low recombination between the alien segments (6S or 6G) and the wheat chromosome 6B [30,31].

It is reported that many of the resistance genes used in agriculture eventually become ineffective with the continual change of the pathogen’s virulence structure [32]. Consequently, there is an urgent need to discover and transfer more powdery mildew resistance genes from alien sources, which represent an abundant genetic resource, to commercial cultivars. The present gene *pmCH89* identified herein was transferred into a commercial wheat background, and a range of powdery mildew resistant introgression lines have been obtained. Henceforth, these lines could be used in wheat breeding programs. Marker data showed that *Xwmc125*, *Xbarc193*, *Xbarc199*, and *Xwmc310* were linked to *pmCH89*. The locations of four linked microsatellite loci were verified with CS nulli-tetrasomic and ditelosomic stocks and deletion lines, and were further confirmed by assigning linked microsatellite markers to chromosome 4BL in the bin 0.68–0.78. Based on the genetic and physical bin maps, the powdery mildew resistance gene *pmCH89* was mapped on the interstitial region of chromosome 4BL and closely flanked by SSR markers *Xwmc310* and *Xwmc125*, which were proximal at 3.1 cM and distal at 2.7 cM, respectively, to the resistance gene. Because resistance to powdery mildew in many Chinese cultivars has been overcome by virulent races of the pathogen, *pmCH89* and the identification of closely flanking markers may be beneficial for increasing the overall diversity of available resistance genes with the potential to provide more comprehensive and durable protection against the disease.

## 4. Materials and Methods

### 4.1. Plant Materials

The materials used in this study were *Th. intermedium* (accession Z1141), and TAI7045, a partial amphiploid developed by crossing common wheat cultivars with *Th. intermedium* accession Z1141. Wheat genotypes CH09W89, Jinchun 5, Jin T2250, Jintai 170, Jinmai 33, and Chinese Spring (CS) were obtained from laboratory germplasm stocks. CS nullisomic-tetrasomic (NT) stocks (N4AT4B, N4BT4D, and N4DT4A), ditelosomic (Dt) 4BS stock, and 4BL deletion lines (4BL-1, 4BL-3, 4BL-4, 4BL-5, 4BL-8, and 4BL-10) were obtained from Dr. B. Friebe, Wheat Genetic and Genomic Resources Center, Kansas State University, Manhattan, KS, USA. CH09W89 is a homogeneous BC_2_F_5_-derived resistant wheat line from Jintai 170/TAI7045//2*Jinmai 33. TAI7045 is the powdery mildew resistance gene donor for CH09W89, which was derived from the cross Jinchun 5/Z1141//Jin T2250.

Wheat cultivars Jingshuang 16, SY95-71, and Mianyang 11 (MY11) are all susceptible to wheat powdery mildew. To study the powdery mildew resistance of CH09W89, we developed segregating populations (F_2_, F_2:3_, and BC_1_) by crossing CH09W89 with susceptible wheat cultivars SY95-71 and MY11. The F_1_ was used for determining the dominance of the resistance and F_2_, F_2:3_, and BC_1_ were tested for segregation of powdery mildew resistance. An F_2_ population and derived F_2:3_ lines from SY95-71/CH09W89 were used for further microsatellite screening and gene mapping. The mapping population was comprised of 175 F_2_ plants and 166 derived F_2:3_ families, the difference being due to insufficient seeds of nine F_2_ plants.

### 4.2. Cytogenetic Analysis

Genomic *in situ* hybridization is a powerful molecular cytogenetic technique to identify alien chromatin in the wheat background. GISH analysis was performed using a similar protocol to that described by Han *et al.* [33]. In this study, *Th. intermedium* Z1141 genomic DNA was used as a probe and the CS genomic DNA was used as blocker DNA.

### 4.3. Evaluation of Powdery Mildew Responses

Seven isolates of the prevailing local *B. graminis* f. sp. *tritici* (*Bgt*) pathotypes provided by the Plant Protection Institute, Chinese Academy of Agricultural Sciences (Table 1), were used for resistance tests in the seedling stage. Using Jingshuang 16 as a control, CH09W89 and the parental lines were grown in 70 × 45 × 18 cm flat plastic trays. When the first leaves were fully expanded, inoculations were performed by dusting conidiospores from sporulating seedlings of Chancellor onto the test seedlings according to the method described by Xiang [34]. Host infection types (ITs) were scored 2–3 weeks after inoculation, when the susceptible check Jingshuang 16 became heavily infected, using a 0–4 rating scale [35], where 0 = no visible symptoms, 0; = necrotic flecks, 1 = necrosis with low sporulation, 2 = necrosis with moderate sporulation, 3 = no necrosis with moderate to high sporulation, and 4 = no necrosis with full sporulation. Scores of 0–2 were classified as resistant and 3–4 as susceptible.

To determine the genetics of resistance in CH09W89, E09, which is a prevalent pathotype in the Beijing area and avirulent on CH09W89, but is virulent on SY95-71 and MY (Mianyang) 11, was used to test F_1_, F_2_, F_2:3_, and BC_1_ populations derived from SY95-71/CH09W89 and CH09W89/MY11//CH09W89 (Table 2). All seeds from the parents, F_1_, F_2_, F_2:3_, and BC_1_ populations were planted in the greenhouse. Twenty seeds of each parent and F_1_, 175 seeds of F_2_, 78 seeds of BC_1_, and 20–25 seeds for each of the F_2_-derived F_2:3_ families were planted randomly in a 100 cm row, 25 cm apart. Susceptible spreaders of Jingshuang 16 and SY95-71 were planted in every 10th row for each population. The predominant *Bgt* race E09 was used for adult plant testing and the spreaders were artificially inoculated two times at the seedling stage. Adult plant reactions were scored twice, at the ear emergence stage and at the milky ripe stage, using the 0–4 scale previously described. To determine the genotypes of F_2_ plants from the cross of CH09W89 with SY95-71, the F_2_-derived F_3_ families were tested with the same race used in the F_2_ tests.

### 4.4. Molecular Marker Analysis

Genomic DNA, extracted from young seedling leaf tissue collected from the F_2_ individuals from which the F_2:3_ families originated, were used for molecular analysis. SSR markers linked to the resistance gene were identified by bulked segregant analysis (BSA). Equal amounts of DNA from 10 resistant F_2_ segregants were pooled into a resistant bulk, and DNA from 10 susceptible F_2_ segregants were pooled into a susceptible bulk according to Michelmore *et al.* [36]. The resistant parent, susceptible parent, resistant bulk (*Br*), and susceptible bulk (*Bs*) were then screened by genome and chromosome specific markers from across the wheat genome. Markers that were polymorphic between the resistant and susceptible parents and bulks were used to genotype the F_2:3_ lines and for linkage analysis.

Wheat microsatellite markers were used to detect polymorphism among parents, and resistant and susceptible bulks. The polymorphism markers were genotyped in F_2_ individuals to determine genetic linkage between the powdery mildew resistance gene(s) and markers.

PCR was performed in a 20 μL solution comprised of 80–100 ng template DNA, 2 μL 10× buffer (10 mM Tris-HCl, pH 8.3, 50 mM KCl, 1.5 mM MgCl_2_), 0.2 mM of each dNTP, 1 unit Taq DNA polymerase, 0.25 μM of each primer. Amplification was performed at 94 °C for 5 min initial denaturation, 35 cycles each consisting of 45 s at 94 °C for denaturation, 45 s at either 50, 55 or 60 °C (based on primer annealing temperature), 1 min at 72 °C for extension; and finally a 10 min extension step at 72 °C before cooling to 4 °C. After PCR amplification, 12 μL of formamide loading buffer (0.4 g/mL sucrose, 1 mg/mL bromophenol blue, and 1 mg/mL xylene cyanol) was added to each PCR product. Then, 4–6 μL of each sample was loaded on 8% non-denaturing polyacrylamide gels (Acr:Bis = 29:1) and separated at 150 V for approximately 2 h, then visualized by silver staining. PCR for each SSR marker was performed in a PTC200 Peltier Thermal Cycler (Bio-Rad Laboratories Inc., Hercules, CA, USA).

### 4.5. Data Analysis and Chromosomal Assignment

The goodness-of-fit of observed phenotypes and expected segregation ratios was determined by χ-square tests (χ^2^). Linkages between markers and the resistance gene were determined using Joinmap 4.0 software [37] with a LOD threshold 3.0. Map distances were determined by using the Kosambi mapping function.

Chromosomal locations of linked microsatellite markers were confirmed by using CS homoeologous group 4 nullitetrasomic, ditelosomic and lines 4BL-4 (FL 0.55), 4BL-3 (FL 0.68), 4BL-1 (FL 0.71), 4BL-8 (FL 0.78), 4BL-5 (FL 0.86), and 4BL-10 (FL 0.95). Markers were located to chromosome bins by determining the smallest deletion bin possessing them.

## 5. Conclusions

A new powdery mildew resistance gene, tentatively designated as *pmCH89*, was found in a putative wheat-*Th. intermedium* introgression line developed by crossing the resistant partial amphiploid TAI7045 with susceptible cultivars, and the resistance is effective against the existing powdery mildew races in China, including the most widely virulent and predominant pathotypes. The gene was physically mapped on the intercalary bin 0.68-0.78 of chromosome 4BL and closely flanked by markers *Xwmc310 and Xwmc125*. *pmCH89*, together with the identified closely linked markers, could be useful in marker-assisted selection for improving powdery mildew resistance in wheat breeding programs.
